# Correlation of C-Peptide Levels With Complications of Type 2 Diabetes Mellitus

**DOI:** 10.7759/cureus.93754

**Published:** 2025-10-03

**Authors:** Nikhil Kumar, Azhar A Khan, Ajay Yadav, Sudheer Yadav, Bechan Kumar Gautam, Rajesh K Rai, Kislay Mishra

**Affiliations:** 1 General Medicine, Baba Raghav Das Medical College Gorakhpur, Gorakhpur, IND; 2 Internal Medicine, Baba Raghav Das Medical College Gorakhpur, Gorakhpur, IND

**Keywords:** c-peptide, diabetic nephropathy, diabetic neuropathy, diabetic retinopathy, macrovascular complications, microvascular complications, type 2 diabetes mellitus, β-cell function

## Abstract

Introduction

Type 2 diabetes (T2DM) is a major worldwide health issue that leads to serious problems in small and large blood vessels. C-peptide, which is released alongside insulin in equal quantities, acts as a reliable indicator of how well the beta cells of the pancreas are functioning and may be associated with diabetes-related complications.

Methodology

This one-year cross-sectional observational study took place at Baba Raghav Das Medical College in Gorakhpur, including 130 patients with type 2 diabetes who had had the condition for more than 10 years. Participants received thorough assessments for small blood vessel issues (such as retinopathy, nephropathy, and neuropathy) and large blood vessel problems (such as coronary artery disease and peripheral vascular disease) using standardized diagnostic criteria. Blood tests measured C-peptide levels, HbA1c, and other lab values. Statistical analysis was conducted using Python 3.12 with appropriate statistical libraries, employing t-tests for continuous variables, chi-square tests for categorical variables, and one-way ANOVA for multi-group comparisons.

Results

The study included 130 participants (n=130, mean age 59.4±12.0 years, 60% female) with a mean diabetes duration of 12.5±2.8 years and a mean HbA1c of 8.5±2.5%. Mean C-peptide level was 6.07±4.61 ng/mL. Microvascular complications showed age-related increases: retinopathy (25% in <45 years to 76.1% in >60 years), nephropathy (12.5% to 47.8%), and neuropathy (12.5% to 58.2%). Patients with complications demonstrated significantly lower C-peptide levels compared to those without complications (retinopathy: 5.29 vs 7.52 ng/mL, t=-2.37, p=0.0208; nephropathy: 4.87 vs 6.77 ng/mL, t=-2.58, p=0.0113; neuropathy: 4.59 vs 7.64 ng/mL, t=-4.12, p=0.0001). High C-peptide levels correlated with better glycemic control, with 48.2% achieving HbA1c <7.5% compared to only 10% in the low C-peptide group (χ²=8.45, p<0.05).

Conclusion

C-peptide levels demonstrate a significant inverse correlation with microvascular complications in T2DM patients. Lower C-peptide concentrations are associated with poorer glycemic control, higher complication rates, and increased insulin dependency, suggesting its potential utility as a biomarker for risk stratification and therapeutic decision-making.

## Introduction

Diabetes mellitus encompasses a spectrum of metabolic disorders characterized primarily by persistent hyperglycemia, resulting from a combination of genetic predisposition, environmental influences, and metabolic dysfunction [1]. The rapidly increasing global burden of type 2 diabetes mellitus (T2DM) has led to its recognition as a modern health crisis, with the International Diabetes Federation reporting approximately 537 million diabetic individuals worldwide, projected to reach 783 million by 2045 [2]. India has emerged as the new "diabetes capital of the world," with recent data from the Indian Council of Medical Research indicating that over 101 million individuals are currently living with diabetes in the country [3].

A significant challenge in T2DM management is that individuals often remain asymptomatic despite poor glycemic control. A considerable proportion of patients already present with either macrovascular complications, such as coronary artery disease or peripheral vascular disease, or microvascular complications, including nephropathy, retinopathy, and neuropathy, by the time of diagnosis [4]. The development of such complications correlates with both the duration of diabetes and the degree of glycemic control [5]. However, even stringent regulation of blood glucose levels does not fully prevent the onset of diabetic complications, highlighting the need for alternative or adjunctive therapeutic strategies and better prognostic markers [6].

C-peptide, an inactive polypeptide with a half-life of approximately 20-30 minutes, is formed during the enzymatic conversion of proinsulin into insulin and is co-secreted with insulin from pancreatic β-cells in equimolar amounts [7]. Its circulating concentration directly reflects the level of endogenous insulin production, making it a valuable biomarker for evaluating pancreatic β-cell function [8]. Among patients with T2DM, insulin levels may vary, ranging from hyperinsulinemia in the context of insulin resistance to hypoinsulinemia due to β-cell failure [9]. The potential association between C-peptide as a proxy for insulin status and the occurrence of either microvascular or macrovascular complications in T2DM remains an under-explored area with promising implications for improving disease management.

Several studies have investigated the relationship between C-peptide levels and diabetic complications with varying results. Recent studies have demonstrated notable associations between C-peptide levels and various diabetic complications, with some suggesting protective roles against microvascular damage [10,11]. Contemporary research has also indicated that C-peptide may serve as both a biomarker and therapeutic agent in metabolic disorders [12].

Given the accessibility and clinical utility of C-peptide measurement, this study aimed to investigate the association between C-peptide levels and the presence of complications in patients with T2DM within the Indian population.

Study objectives

Primary Objective

To investigate the correlation between serum C-peptide levels and diabetic complications (both microvascular and macrovascular) in patients with type 2 diabetes mellitus.

Secondary Objectives

To assess the relationship between C-peptide levels and glycemic control, to evaluate the association between C-peptide levels and treatment requirements, and to analyze age-related patterns of complications across different C-peptide categories.

## Materials and methods

Study design

This cross-sectional observational study was conducted to investigate the association between C-peptide levels and complications in type 2 diabetes. It was carried out in the Department of Medicine at BRD Medical College in Gorakhpur, Uttar Pradesh, over one year (January 2024 to December 2024). Ethical clearance was granted by the Institutional Ethics Committee of Baba Raghav Das Medical College, Gorakhpur, with reference number 175/IHEC/2024.

Study population

The study enrolled 130 patients with established T2DM who presented to the inpatient department settings at our centre. The sample size was determined using data from a previous study reporting a pooled prevalence of diabetes mellitus in India at 22% [13]. Power analysis indicated that 130 patients would provide 80% power to detect a clinically significant difference in C-peptide levels between patients with and without complications (effect size = 0.5, α = 0.05).

Inclusion criteria

Inclusion criteria comprised all patients with type 2 diabetes mellitus of more than 10 years duration (to ensure adequate exposure time for complication development), with or without complications, aged between 18-65 years (to exclude age-related confounding factors in elderly patients). Written informed consent was obtained from all participants after a detailed explanation of the study methodology, associated risks, and benefits in their preferred language.

Exclusion criteria

Exclusion criteria included pregnant women, patients with secondary diabetes mellitus, subjects on steroids or undergoing dialysis, patients with type 1 diabetes mellitus, and those with acute diabetic emergencies.

Methodology

Diabetic Retinopathy Assessment

Evaluation for diabetic retinopathy was performed through direct ophthalmoscopy by qualified ophthalmologists using standardized protocols. We acknowledge that indirect ophthalmoscopy and fundus photography are gold standards; however, direct ophthalmoscopy was used due to resource constraints at our institution. Retinopathy was classified according to the International Clinical Diabetic Retinopathy Disease Severity Scale [14]: no apparent retinopathy, mild non-proliferative diabetic retinopathy (NPDR), moderate NPDR, severe NPDR, and proliferative diabetic retinopathy (PDR).

Diabetic Nephropathy Assessment

Diabetic nephropathy was classified based on KDIGO (Kidney Disease: Improving Global Outcomes) guidelines using estimated glomerular filtration rate (eGFR) and albuminuria levels [15]: Stage 1: eGFR ≥90 mL/min/1.73m² with albuminuria, Stage 2: eGFR 60-89 mL/min/1.73m² with albuminuria, Stage 3a: eGFR 45-59 mL/min/1.73m², Stage 3b: eGFR 30-44 mL/min/1.73m², Stage 4: eGFR 15-29 mL/min/1.73m², and Stage 5: eGFR <15 mL/min/1.73m².

Diabetic Neuropathy Assessment

Diabetic neuropathy was diagnosed through clinical examination using the Michigan Neuropathy Screening Instrument (MNSI) [16] and nerve conduction studies, where indicated. MNSI scores ≥2.5 were considered indicative of neuropathy. Assessment included vibration sensation using a 128-Hz tuning fork, light touch sensation using a 10-g monofilament, ankle reflexes, and symptom assessment questionnaire.

Macrovascular Complications Assessment

Coronary artery disease assessment was based on clinical history, previous medical records, 12-lead electrocardiogram findings, and echocardiography, where available. We acknowledge that coronary angiography is the gold standard; however, our assessment used non-invasive methods due to resource limitations. Criteria included previous myocardial infarction documented in medical records, typical anginal symptoms with ECG changes, and previous revascularization procedures. Peripheral arterial disease assessment included clinical examination for claudication pain, absent pulses, and the presence of ulcers. Doppler studies were performed when clinically indicated due to resource constraints.

Laboratory methods

C-peptide Assay

Fasting serum C-peptide levels were measured using chemiluminescent immunoassay (CLIA) on an automated analyzer (Siemens Advia Centaur). Blood samples were collected after 8-12 hours of fasting, and serum was separated within 30 minutes of collection. The assay had an analytical sensitivity of 0.05 ng/mL and a coefficient of variation <5%.

Other Laboratory Parameters

The comprehensive investigation panel included complete blood count, HbA1c (measured by high-performance liquid chromatography), lipid profile including triglycerides and LDL cholesterol, urine albumin (by immunoturbidimetric method), serum creatinine, and C-reactive protein levels.

Reference ranges and cut-offs

The following reference ranges and cut-offs were used for laboratory parameters: fasting C-peptide normal range was 1.1-4.4 ng/mL, HbA1c target for diabetes was <7.0%, C-reactive protein normal range was <3.0 mg/L, serum creatinine normal range was 0.7-1.3 mg/dL for males and 0.6-1.1 mg/dL for females, and urine albumin normal range was <30 mg/g creatinine.

C-peptide Categorization: For the purpose of this study, C-peptide levels were categorized as follows: high C-peptide was defined as >4.4 ng/mL, normal C-peptide ranged from 1.1-4.4 ng/mL, and low C-peptide was defined as <1.1 ng/mL.

Statistics

Statistical analysis was conducted using Python 3.12 with libraries including Pandas for data handling, SciPy for statistical tests, and stats models for regression modelling. Data were presented as means with standard deviations for continuous variables (Mean±SD) and as percentages (%) for categorical ones. Independent samples t-tests were used to compare mean C-peptide levels between patients with and without complications, with t-values reported alongside p-values. The Chi-square test from SciPy was used to compare categorical variables with chi-square values reported. For comparisons across more than two groups, one-way ANOVA from SciPy or stats models was applied, with F-values reported. Odds ratios were derived via univariate and multivariable logistic regression in Stats models with corresponding z-statistics reported. Statistical significance was set at p<0.05, with highly significant results considered at p<0.001.

## Results

The study included 130 people with type 2 diabetes (n = 130), with an average age of 59.4 ± 12.0 years (Table 1). More women participated, with 78 females (60.0%) versus 52 males (40.0%). The average time since diabetes diagnosis was 12.5 ± 2.8 years, showing a group with long-term disease. Blood sugar control was not ideal, with an average HbA1c of 8.5 ± 2.5%. Average C-peptide levels were 6.07 ± 4.61 ng/mL, suggesting wide differences in beta-cell function in the pancreas. For treatment, 99 patients (76.2%) used oral medications to lower blood sugar, while 31 (23.8%) needed insulin.

**Table 1:** Baseline Demographic and Clinical Characteristics (n=130) Mean±SD for continuous variables, n (%) for categorical variables OHA: oral hypoglycemic agents

Parameter	Value
Number of Patients (n)	130
Age (years), mean ± SD	59.4 ± 12.0
Gender - Male	52 (40.0%)
Gender - Female	78 (60.0%)
Duration of Diabetes (years), mean ± SD	12.5 ± 2.8
HbA1c (%), mean ± SD	8.5 ± 2.5
C-Peptide (ng/mL), mean ± SD	6.07 ± 4.61
Treatment - OHA	99 (76.2%)
Treatment - Insulin	31 (23.8%)

The distribution of diabetic complications across different age groups revealed significant patterns (Table 2). Microvascular complications demonstrated a clear age-related increase. Retinopathy prevalence increased substantially from 25.0% in patients under 45 years to 76.1% in those over 60 years. Similarly, nephropathy showed progression from 12.5% in the youngest group to 47.8% in the oldest group. Neuropathy followed a comparable pattern, increasing from 12.5% to 58.2% across age groups. In contrast, macrovascular complications represented by coronary artery disease and peripheral vascular disease remained relatively stable across age groups, ranging from 25.0% to 28.4%.

**Table 2:** Distribution of Diabetic Complications Among Different Age Groups (n=130) Values presented as n (%). CVD/PVD: coronary artery disease/peripheral vascular disease

Age Group	Retinopathy	Nephropathy	Neuropathy	CAD/PVD
<45 (n=21)	25.0%	12.5%	12.5%	25.0%
45–60 (n=45)	63.8%	29.8%	55.3%	27.7%
>60 (n=64)	76.1%	47.8%	58.2%	28.4%

The connection between C-peptide levels and blood sugar management showed notable patterns (Table 3). Those with high C-peptide had the best control, with 40 people (48.2%) reaching HbA1c below 7.5%. This fell to 12 people (32.4%) in the normal C-peptide group and just one person (10.0%) in the low group. In contrast, poor control (HbA1c above 8.5%) was most common in the normal group (48.6%) and low group (40.0%), compared to 37.3% in the high group.

**Table 3:** Distribution of HbA1c Categories Across Different C-Peptide Categories (n=130) Values presented as n (%), χ²=8.45, p<0.05.

C-Peptide Category	<7.5	7.5–8.5	>8.5
High (n=83)	40 (48.2%)	12 (14.4%)	31 (37.3%)
Normal (n=37)	12 (32.4%)	7 (18.9%)	18 (48.6%)
Low (n=10)	1 (10.0%)	5 (50.0%)	4 (40.0%)

Among patients achieving good glycaemic control (HbA1c ≤7.5%), significant differences in complication rates were observed across C-peptide categories (Table 4). In the high C-peptide group, retinopathy affected 19 patients (47.5%), neuropathy, 15 patients (37.5%), nephropathy, 15 patients (37.5%), and coronary artery disease or peripheral vascular disease, 8 patients (20.0%). The single patient in the low C-peptide group with good glycaemic control demonstrated 100% prevalence of both retinopathy and neuropathy, highlighting the persistent risk associated with β-cell dysfunction even with optimal glucose control.

**Table 4:** Comparison of Diabetic Complications in Patients With HbA1c ≤7.5% According to C-Peptide Category (n=53) Values presented as n (%).

C-Peptide Category	Retinopathy	Neuropathy	Nephropathy	CAD/PVD	OHA	Insulin
High (n=40)	19 (47.5%)	15 (37.5%)	15 (37.5%)	8 (20.0%)	20 (50.0%)	20 (50.0%)
Normal (n=12)	5 (41.7%)	3 (25.0%)	2 (16.7%)	3 (25.0%)	7 (58.3%)	5 (41.7%)
Low (n=1)	1 (100.0%)	1 (100.0%)	0 (0.0%)	0 (0.0%)	0 (0.0%)	1 (100.0%)

Analysis comparing average C-peptide levels in patients with and without certain complications found strong ties to small blood vessel issues (Table 5). Those with retinopathy had notably lower average C-peptide than those without (5.29 vs 7.52 ng/mL, t=-2.37, p=0.0208). Similarly, nephropathy patients showed lower levels (4.87 vs 6.77 ng/mL, t=-2.58, p=0.0113). The largest gap was in neuropathy, with affected patients having much lower C-peptide (4.59 vs 7.64 ng/mL, t=-4.12, p=0.0001). However, large blood vessel complications showed no meaningful difference (5.89 vs 6.13 ng/mL, t=-0.32, p=0.7476).

**Table 5:** Comparison of Mean C-Peptide Levels in Patients With and Without Diabetic Complications Mean±SD ng/mL, statistical significance tested using independent samples t-test

Complication	Present Mean C-Peptide (ng/mL)	Absent Mean C-Peptide (ng/mL)	t-value	p-value	Significant (p<0.05)
Retinopathy	5.29	7.52	-2.37	0.0208	Yes
Nephropathy	4.87	6.77	-2.58	0.0113	Yes
Neuropathy	4.59	7.64	-4.12	0.0001	Yes
CAD/PVD	5.89	6.13	-0.32	0.7476	No

The link between diabetes duration and complications, grouped by C-peptide levels, revealed key trends (Table 6). Among those with 10-15 years of diabetes, patients with low C-peptide had higher neuropathy rates (100%) than those with high C-peptide (32.0%). For the 15-20 year group, low C-peptide had a bigger impact, with 100% experiencing both retinopathy and neuropathy, compared to 81.8% retinopathy and 63.6% neuropathy in the high C-peptide group.

**Table 6:** Correlation of C-Peptide and Duration of Diabetes With Complications (n=130) Values presented as n (%)

Duration	C-Peptide Category	Retinopathy	Nephropathy	Neuropathy	CAD/PVD
10-15 years (n=74)	High (n=50)	26 (52.0%)	9 (18.0%)	16 (32.0%)	13 (26.0%)
	Normal (n=20)	9 (45.0%)	4 (20.0%)	8 (40.0%)	4 (20.0%)
	Low (n=4)	3 (75.0%)	0 (0.0%)	4 (100.0%)	1 (25.0%)
15-20 years (n=56)	High (n=33)	27 (81.8%)	21 (63.6%)	21 (63.6%)	11 (33.3%)
	Normal (n=17)	14 (82.4%)	11 (64.7%)	12 (70.6%)	5 (29.4%)
	Low (n=6)	6 (100.0%)	3 (50.0%)	6 (100.0%)	2 (33.3%)

## Discussion

This comprehensive cross-sectional study examined the association between C-peptide levels and complications in 130 patients with type 2 diabetes, offering key insights into how beta-cell function influences disease outcomes. Results demonstrated strong associations between lower C-peptide and greater risk of small blood vessel complications, along with different patterns for large blood vessel disease. The demographic characteristics of our study population, with a mean age of 59.4 years and female predominance (60%), reflect typical presentations of established T2DM in clinical practice. The mean diabetes duration of 12.5 years and suboptimal glycaemic control (mean HbA1c 8.5%) align with real-world diabetes management challenges [17]. The wide variation in C-peptide levels (6.07±4.61 ng/mL) underscores the heterogeneous nature of β-cell function in T2DM patients, which has important therapeutic implications. The age-related increase in microvascular complications observed in our study corroborates established epidemiological patterns. The progression of retinopathy from 25% in patients under 45 years to 76.1% in those over 60 years reflects the cumulative impact of chronic hyperglycaemia and duration of exposure [18]. Similarly, the increase in nephropathy and neuropathy with advancing age aligns with natural history studies of diabetic complications [19]. The relatively stable prevalence of macrovascular complications across age groups suggests different pathophysiological mechanisms compared to microvascular disease.

Our results demonstrate a clear association between higher C-peptide levels and improved blood sugar management, as 48.2% of patients with elevated C-peptide reached target HbA1c levels, compared to just 10% in the low C-peptide group. This association suggests maintained beta-cell function that supports better glucose balance and lower insulin resistance [20]. The inverse relationship between C-peptide levels and poor glycaemic control reinforces the concept that beta-cell dysfunction plays a central role in diabetes progression and treatment failures.

A major finding was the strong association between lower C-peptide and small blood vessel complications. Individuals with retinopathy, nephropathy, and neuropathy had notably lower average C-peptide levels than those without these issues. The statistical significance of these findings, as demonstrated by the t-test results (retinopathy: t=-2.37, p=0.0208; nephropathy: t=-2.58, p=0.0113; neuropathy: t=-4.12, p=0.0001), provides robust evidence for the inverse relationship between C-peptide levels and microvascular complications. The largest difference appeared in neuropathy (4.59 vs 7.64 ng/mL, p=0.0001), suggesting that peripheral nerve function could be especially vulnerable to beta-cell impairment. These outcomes align with prior studies suggesting C-peptide offers direct protection to small blood vessels, going beyond its function as an insulin indicator [21].

The absence of significant association between C-peptide levels and macrovascular complications in our study (t=-0.32, p=0.7476) contrasts with some previous reports suggesting increased cardiovascular risk with elevated C-peptide levels [22]. This discrepancy may reflect the complex relationship between insulin resistance, β-cell function, and cardiovascular disease, where elevated C-peptide may indicate compensatory hyperinsulinemia rather than preserved function in the context of severe insulin resistance [23]. The relatively stable prevalence of macrovascular complications across C-peptide categories suggests that cardiovascular risk in T2DM is influenced by multiple factors beyond β-cell function.

The analysis of patients with good glycaemic control (HbA1c ≤7.5%) revealed that even among well-controlled individuals, those with low C-peptide levels had higher complication rates. This observation suggests that β-cell function may independently influence complication risk beyond glycaemic control, possibly through direct effects of C-peptide on vascular and neural health [24]. The finding has important implications for understanding the multifactorial nature of diabetic complications and the limitations of glucose-centric management approaches.

The relationship between disease duration and complications stratified by C-peptide levels demonstrated the progressive nature of β-cell dysfunction and its consequences. Patients with longer diabetes duration (15-20 years) and low C-peptide levels showed near-universal prevalence of microvascular complications, highlighting the cumulative impact of β-cell failure over time. The protective effect of preserved C-peptide levels was evident even in patients with prolonged disease duration, suggesting that strategies to preserve β-cell function may have long-term benefits [25].

Our study found a clear association between low C-peptide levels and the need for insulin (all patients with low C-peptide required it), highlighting how measuring C-peptide can help in choosing treatments. This aligns with existing guidelines that recommend checking C-peptide to decide when to start insulin in type 2 diabetes patients [26]. The association between beta-cell function and treatment needs emphasizes the value of personalized care based on remaining insulin production ability.

Our research has several limitations worth noting. The cross-sectional design prevents establishment of causal relationships between C-peptide levels and complications. C-peptide measurement at a single time point might not reflect true long-term beta-cell function, since levels can vary due to factors like recent meals or current blood glucose [27]. While the sample size was adequate for detecting main associations, it might lack sufficient power for detailed subgroup analyses. Additionally, recruitment from a single center may limit generalizability to diabetes patients in other settings [28].

The use of direct ophthalmoscopy for retinopathy assessment, while performed by qualified ophthalmologists, represents a limitation as indirect ophthalmoscopy and fundus photography are considered gold standards. Our CAD assessment relied on non-invasive methods rather than coronary angiography, and Doppler studies for peripheral vascular disease were performed only when clinically indicated due to resource constraints. These methodological limitations should be considered when interpreting our findings. The clinical implications of our findings are significant for diabetes management. C-peptide measurement could serve as a valuable biomarker for risk stratification, helping identify patients at higher risk for microvascular complications who may benefit from more intensive monitoring and intervention. The strong association between low C-peptide and insulin requirement suggests that C-peptide levels could guide therapeutic decisions, potentially leading to earlier insulin initiation in appropriate patients. Furthermore, the findings support the concept that preserving β-cell function should be a key therapeutic goal in T2DM management.

Future research should focus on longitudinal studies to establish causal relationships between declining C-peptide levels and complication development. Investigation of therapeutic interventions aimed at preserving β-cell function and their impact on complication rates would provide valuable clinical evidence. Development of standardized C-peptide thresholds for clinical decision-making and risk stratification would enhance the practical utility of these measurements in routine diabetes care.

## Conclusions

This research demonstrates strong inverse associations between C-peptide levels and small blood vessel complications in individuals with type 2 diabetes. Decreased C-peptide was associated with worse blood sugar management, increased instances of retinopathy, nephropathy, and neuropathy, as well as greater need for insulin treatment. The robust statistical evidence, demonstrated through t-test analyses showing significant differences in mean C-peptide levels between patients with and without microvascular complications (all p-values <0.05), supports the potential utility of C-peptide testing as a valuable indicator for assessing risks and making treatment decisions in managing type 2 diabetes.

The age-related increase in microvascular complications, combined with the protective effects of preserved C-peptide levels, highlights the importance of β-cell function in determining long-term diabetic outcomes. Even among patients achieving target glycaemic control, those with reduced C-peptide levels demonstrated higher complication rates, suggesting that β-cell function independently influences vascular health beyond glucose homeostasis. This research supports using C-peptide tests to identify patients at higher risk for complications, who might benefit from closer monitoring and earlier treatments. The clear association between low C-peptide levels and the need for insulin provides helpful guidance for treatment choices. These results improve our understanding of the complex relationship between beta-cell function and diabetes complications, highlighting the value of approaches that preserve the body's own insulin production.

Future longitudinal studies are needed to establish causal relationships and develop evidence-based therapeutic interventions targeting β-cell preservation. The development of standardized C-peptide thresholds for clinical decision-making would enhance the practical application of these findings in routine diabetes care, potentially leading to improved outcomes through personalized management approaches.
